# Blockade of connexin43-containing hemichannel attenuates the LPS-induced inflammatory response in human dental pulp cells by inhibiting the extracellular flux of ATP and HMGB1

**DOI:** 10.3389/froh.2024.1496819

**Published:** 2024-12-02

**Authors:** Peiling Hu, Ping Long, Ruotong Li, Xiaorong Lan, Yuanpei He, Guangwen Li, Shiting Li

**Affiliations:** 1 School of Stomatology, Southwest Medical University, Lu Zhou, China; 2 Luzhou Key Laboratory of Oral & Maxillofacial Reconstruction and Regeneration, The Affiliated Stomatological Hospital, Southwest Medical University, Luzhou, China

**Keywords:** deep caries, connexin43 hemichannel, inflammatory response, dental pulp cells, DAMPs

## Abstract

**Introduction:**

Tissue repair can be promoted by moderate inflammation but suppressed by excessive levels. Therefore, control of excessive inflammation following removal of infection plays a critical role in promotion of pulpal repair. Connexin 43 (Cx43) forms hemichannels (HCs) or gap channels (GJs) to facilitate the delivery of small molecules between cells to regulate both inflammation and repair. Understanding the role of Cx43 in dental pulp may help develop a potential strategy to attenuate the inflammation and promote the formation of reparative dentin in deep caries.

**Methods:**

We firstly investigated the expression profile of Cx43 in infected human third molars by histological analysis; then, we detected channel activity of Cx43 and the effect of mediating release of small molecules in lipopolysaccharide (LPS)-induced inflammation in human dental pulp cells (hDPCs) by molecular biology methods. Results were analyzed by one-way ANOVA and the unpaired *t*-test. The level of significance was set at *α* = 0.05.

**Results:**

Analysis showed that the expression of Cx43 was upregulated in human third molars as the degree of infection increased, and Cx43 was not only expressed in odontoblast layer, but also detected in cell-rich zone and pulp proper. LPS activated Cx43 HCs in hDPCs while inhibiting GJs; blockade of Cx43 HCs attenuated LPS-induced inflammation. Furthermore, LPS promoted the extracellular release of adenosine triphosphate (ATP) and high-mobility group box 1 (HMGB1) within hDPCs, thus exacerbating LPS-induced inflammation. The blockade of Cx43 HCs inhibited the extracellular release of ATP and HMGB1 within hDPCs.

**Conclusion:**

Collectively, our finding suggested that Cx43 plays a key role in infection and inflammation in dental pulp. LPS activates Cx43 HCs to mediate the extracellular release of ATP and HMGB1 to exacerbate LPS-induced inflammation of hDPCs.

## Introduction

Dental caries is a common oral disease characterized by the chronic and progressive destruction of hard dental tissues under the influence of multiple factors, mainly bacteria. With disease progression, bacterial components and metabolites diffuse within the dentinal tubules, gradually invading the dental pulp. Odontoblasts and dental pulp cells are known to detect infection within the dentin and release autocrine and paracrine signaling factors to combat infection (1–3), including antimicrobial peptides to kill invading microorganisms, and chemokines and cytokines to activate the inflammatory response of dental pulp and eliminate foreign substances (4–6). During the early stages of infection, odontoblasts participate in sensing the inflammatory environment and regulating the innate immune response. In later stages, pulp cells, endothelial cells, and resident immune cells in pulp tissues are activated to respond to bacterial infection (7, 8). The outcome of infection depends on the balance between inflammation and regeneration (9, 10). If infection is not cleared in time, the infected host cells begin to release a large number of inflammatory factors. This process can lead to overactivation of inflammation, thus causing further damage to the dental pulp, and eventually, necrosis of the pulp tissues (11). Mild inflammation or early infection control can maintain cytokines at relatively low levels, and pro-inflammatory factors, such as lipopolysaccharide (LPS), can interact with Toll-like receptor (TLR)-4 to induce the osteogenic/odontoblastic differentiation of pulp cells via mitogen-activated protein kinase (MAPK) and Nuclear factor kappa B (NF-κB) signaling pathways (12, 13). These finding suggest that there is a close relationship between inflammatory and reparative responses in the dental pulp. Only when excessive inflammation is controlled after the removal of infection, can the repair and regeneration of pulp tissues occur. However, how to control the excessive inflammation is not fully understood.

Connexin 43 (Cx43) is one of the most common and abundant forms of connexin (14, 15) and forms gap junctions (GJs) or hemichannels (HCs) that mediate the direct transfer of substances with a molecular weight of <1.5 kDa [such as adenosine triphosphate (ATP) and Ca^2+^] between adjacent cells or between the intracellular and extracellular environments to regulate physiological and pathological activities in a variety of tissues and cells (16, 17). Moreover, Cx43 is known to play a crucial role in maintaining balance between the intracellular and extracellular environments by virtue of its channel activity (18–20). Cx43 is involved in the inflammatory response in a variety of tissues and organs and plays a positive role in tissue repair (21–23). Previous research showed that Cx43 is expressed at high levels in the odontoblast layer during the formation of dentin, thus indicating that Cx43 is associated with odontoblast differentiation and mineralization (24–27). In our previous study, we confirmed that Cx43 plays a role in the odontoblastic differentiation of human dental pulp cells (hDPCs), potentially via the mediation of GJs (28). Recent research identified changes in the expression levels of Cx43 in infected pulp tissue, although different opinions have been reported in relation to how the expression profile changes (27, 29, 30). Two factors may be able to explain these contradictory findings. First, different degrees of damage to carious teeth of clinical origin may lead to varying degrees of pulpal response, secondly, the pulp may have been experiencing different stages of inflammatory and reparative responses. Nevertheless, these results suggested that Cx43 was involved in the infectious and inflammatory response of dental pulp. However, the role of Cx43 in the inflammatory response of dental pulp has yet to be identified.

In the present study, we compared the expression profiles of Cx43 in human teeth with superficial/intermediate caries, deep caries and pulpitis, and investigated the effect of Cx43 channel activity and its mediatory molecules on the LPS-induced inflammatory response of hDPCs. Our goal was to determine the specific role of Cx43 in the response of dental pulp tissues to infection and inflammation.

## Methods

### Reagents

The main experimental reagents used in this study included LPS from Sigma (USA), reverse transcription kit and SYBR Premix Ex TaqTM Kit from Novagen (China), phosphorylated NF-κB antibody (p-NF-κB) from CST (USA), NF-κB antibody from Abcam (USA), Toll-like receptor 4 (TLR-4) from Santa (USA), Gap19, Gap26, and HMGB1 from MCE (USA), ATP reagent and ATP detection kit from Bi Yun Tian (China), ELISA kit from Wuhan Sevier (China), and ethidium bromide (EB) and LY from Sigma (USA).

### Histological analysis

This study was approved by the Ethics Committee of the Affiliated Stomatology Hospital of Southwest Medical University, approval number [20180511001]. The human third molars diagnosed with superficial/intermediate caries, deep caries, reversible pulpitis, and irreversible pulpitis through clinical and radiological examinations were selected for this experiment, with 3 samples in each group. The impacted teeth were collected from adults and informed consent was obtained from all participants. The teeth were fixed in 4% paraformaldehyde solution for 48 h, followed by decalcification in 10% EDTA (pH 7.4) at room temperature for 12 months. For light microscope analyses, tissues were embedded in paraffin. Thin sections (4 µm) were stained with hematoxylin and eosin or processed for immunofluorescence analysis.

### Cell culture

Healthy and intact premolars were extracted for orthodontic treatment purposes at the Stomatology Hospital of Southwest Medical University under approved guidelines by the Ethics Committee of Southwest Medical University. All patients provided informed consent. hDPCs were isolated and prepared as described previously (31). Cells were maintained in α-minimal essential medium (Thermo Fisher Scientific Inc.) with 10% fetal bovine serum (Thermo Fisher Scientific Inc.) and 1% penicillin/streptomycin (Thermo Fisher Scientific Inc.) at 37°C in a humidified atmosphere of 95% air and 5% CO_2_. Cells from passages 3–10 were used for all experiments. hDPCs were stimulated with 5 µg/ml LPS for 6 h. In subsequent experiments, the cells were incubated with 2 μM Gap19 TFA for 30 min, 50 μM Gap26 for 1.5 h, 5 mM ATP for 6 h, and 1 µg/ml HMGB1 for 24 h. Results arising from concentration screening are shown in Supplementary Figure S1.

### Immunofluorescence (IF)

Tissue slices or cell samples were permeabilized with 0.1% Triton X-100 for 15 min, followed by blocking with 1% BSA for 1 h. Sections were then incubated with Cx43 (1:1000) or HMGB1 (1:50) at 4°C overnight. The following morning, the sections were incubated with ALEXAFluor 488/647-conjugated secondary antibodies (1:300, ab150077/ab150115; Abcam) for 1.5 h, and subsequently counterstained with DAPI (Solarbio, Beijing, China) for 10 min. Finally, the samples were imaged using a LEICA DM4000B microscope equipped with a Photometrics CoolSnap1 camera and corresponding software (Leica).

### Short hairpin RNA gene knockdown

pLKD-CMV-EGFP-2A-Puro-U6 vectors containing Cx43/GJα1 and negative control (mock) short hairpin RNA (shRNA) sequences were purchased from OBiO Technology Company (Shanghai, China). The Cx43/GJα1 and mock shRNA sequences were 5′-CCTGGCTCATGTGTTCTAT-3′ and 5′-TTCTCCGAACGTGTCACGT-3′, respectively. hDPCs were transfected with shRNA-Cx43 and shRNA-mock lentiviral particles for 12 h, followed by 1 μg/ml puromycin selection for 3 days to acquire cells that were stably transfected with lentiviral particles. The results were shown in Supplementary Figure S2.

### Real-time quantitative polymerase chain reaction (qRT-PCR)

Total RNA was extracted from cultured cells and first-strand cDNA was synthesized from 0.5 µg of RNA. Gene expression was analyzed by qRT-PCR with an Applied Biosystems (Foster, CA) 7500 Real-time PCR System in a total volume of 20 µl containing 10 mmol/L each of specific primers (Table 1). Levels were standardized to the housekeeping gene *GAPDH* and expressed as relative mRNA levels.

**Table 1:** Gene-specific primers for PCR amplification.

Gene	Primers (5′-3′)
GAPDH	F: ATGGGGAAGGTGAAGGTCG
	R: GGGGTCATTGATGGCAACAATA
IL-1β	F: GCCAGTGAAATGATGGCTTATT
	R: AGGAGCACTTCATCTGTTTAGG
IL-6	F: CACTGGTCTTTTGGAGTTTGAG
	R: GGACTTTTGTACTCATCTGCAC
IL-8	F: ACTTTCAGAGACAGCAGAGCACAC
	R: CACACAGTGAGATGGTTCCTTCCG
TNF-α	F: AAGGACACCATGAGCACTGAAAGC
	R: AGGAAGGAGAAGAGGCTGAGGAAC
Cx43	F: CTGGGGGTGTATGGGGTAGA
	R: TTCTTAGGGGTGTTTGCGGG
HMGB1	F: GAACAACACTGCTGCGGATG
	R: TCCTCCTCGTCGTCTTCCTC

### Western blotting (WB)

Total protein was isolated in RIPA buffer (R0278, Sigma-Aldrich), separated by SDS-PAGE, and transferred to PVDF membranes (EMD Millipore). Subsequently, the membranes were incubated with a blocking buffer containing 5% non-fat dried milk in TBS Tween-20 buffer and cultured at 4°C overnight with antibodies against NF-κB, p-NF-κB (1:1000), TLR4 (1:1000), and Cx43 (1:1000), followed by incubation with secondary antibodies at 37°C for 2 h. Blots were developed with a Supersignal West Pico chemiluminescent substrate kit (Pierce) with GAPDH serving as a loading control.

### Ethidium bromide (EtBr) dye uptake experiment

hDPCs were seeded into confocal culture dishes at a density of 1.5 × 10^4^ cells per well. Subsequently, each well was treated with 5 μM EtBr staining solution and incubated at 37°C in the dark for 10 min. The cells were then fixed with 4% paraformaldehyde for 10 min, and observed and imaged under an inverted fluorescence microscope.

### LY scratch labeling dye tracing experiment

hDPCs were cultured in a 6-well plate at a density of 1.5 × 10^4^ cells per well. Subsequently, 1 ml of 0.5% LY fluorescent dye was added. 3–4 straight scratches were made using a sharp scalpel, and the cells were then incubated in a 37℃ incubator for 10 min in the dark. After removing the dye, the cells were fixed with 4% paraformaldehyde for 10–15 min and finally observed and imaged under an inverted fluorescence microscope.

### Enzyme-linked immunosorbent assay (ELISA)

The protein levels of IL-1β, IL-8, and HMGB1 were investigated using commercially available enzyme-linked immunosorbent assay kits according to the manufacturer's instructions (GEH0002 and GEH0005 for IL-1β and IL-8, Wuhan Sevier Biotechnology Co., Ltd.; HM10235 for HMGB1, Bioswamp Biotechnology Co., Ltd.).

### Measurement of ATP production

ATP production was measured using an ATP detection kit in accordance with the manufacturer's instructions (Beyotime, China). In brief, the culture supernatant of cells was collected and mixed with ATP standard solutions; then, relative fluorescence was detected by a microplate reader (Infinite M200, TECAN, Vienna, Austria). Finally, the concentration of ATP was calculated according to a standard curve.

### Statistical analysis

Values are expressed as the mean ± standard deviation (SD). Statistical analysis was carried out with SPSS version 20.0 and GraphPad Prism version 9, and one-way analysis of variance (ANOVA) and unpaired *t*-test were used to analyze the data. The results of all experiments represent those of three biological replicates. The mean fluorescence intensity was quantified using ImageJ software (NIH), and subsequent statistical analysis was performed using GraphPad. The data were analyzed by one-way ANOVA, and a *P*-value < 0.05 was considered statistically significant.

## Results

### Analysis of the expression profile of Cx43 in infected human teeth

**Figure 1:**
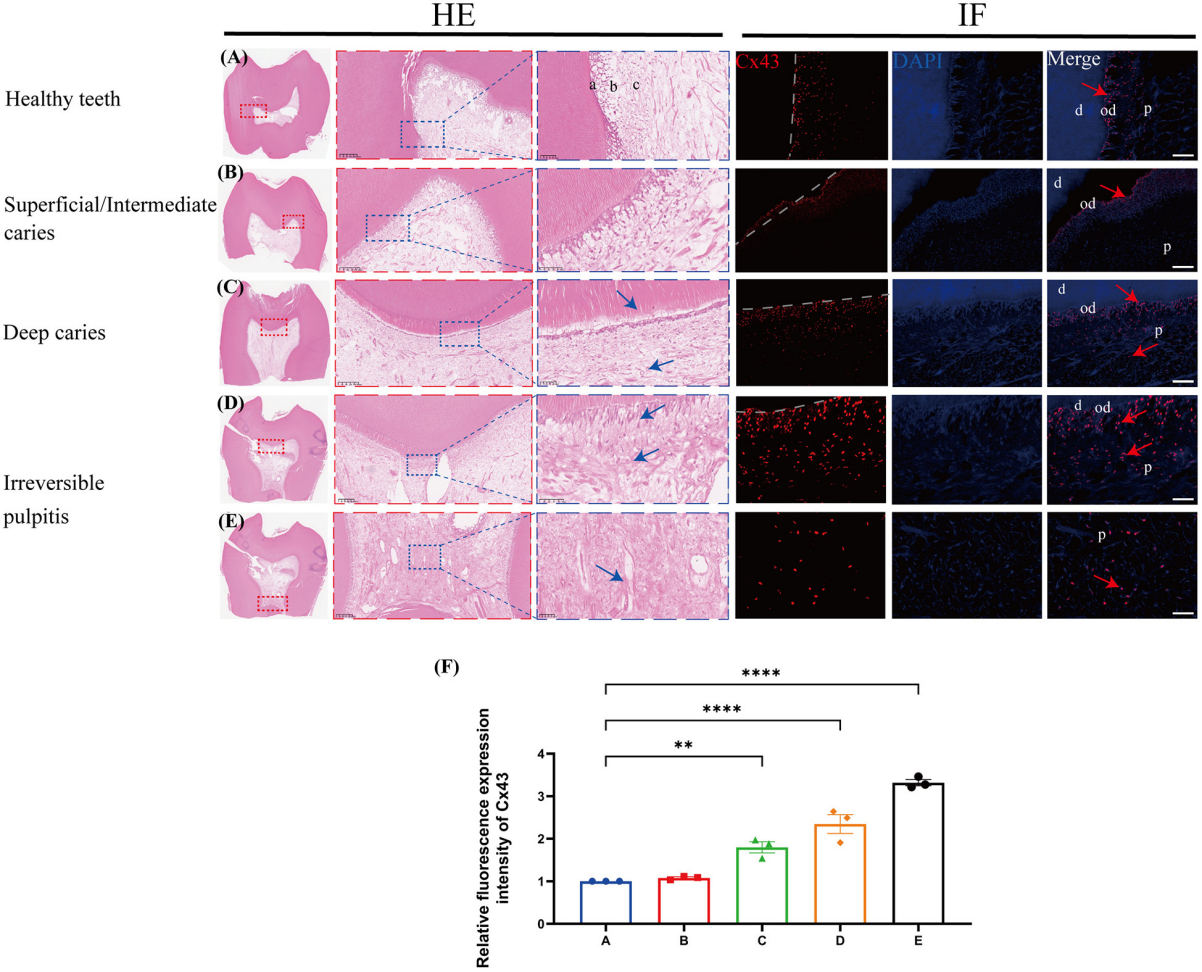
The expression profile of C×43 in human third molars (*n* = 3/group). C×43 (red arrow) immunofluorescence staining and quantitative analysis in infected teeth and healthy controls. Blue arrows: inflammatory cells; a/od: odontoblast layer; b: cell-rich zone; c: pulp proper; d: dentin; P: dental pulp; **(A)** Healthy teeth; **(B)** Superficial/Intermediate caries; **(C)** Deep caries; (D,E) Irreversible pulpitis; scale bar: 20 μm; **(F)** Quantitative fluorescence analysis of C×43 in various groups of dental pulp. **P < 0.01, ****P < 0.0001.

In healthy teeth, the dental pulp structure was complete and uniform, with a palisade-like arrangement in the odontoblast layer, a cell-free zone immediately adjacent to the odontoblast layer, and a densely cell-rich zone beneath it (Figure 1A). The pulp cells were evenly distributed throughout the pulp proper, and Cx43 was only weakly expressed in the odontoblast layer. In superficial/intermediate caries, the expression of Cx43 in the odontoblast layer was significantly increased (Figure 1B). As the lesion gradually progressed (Figure 1C,D), the dentin was destroyed and disintegrated, leading to the loss of the original odontoblast morphology. Beneath the caries, the infiltration of inflammatory cells in the pulp tissue gradually increased. In addition, the expression of Cx43 was gradually upregulated, with a small amount of Cx43 detected in the cell-rich zone. In cases of irreversible pulpitis (Figure 1E,F), we observed vacuolar degeneration of the odontoblasts. Furthermore, the pulp became edematous with a significant infiltration of inflammatory cells, leading to the formation of multiple small abscesses. At this time, Cx43 was not only strongly expressed below the dentin but also present in the pulp proper, showing a gradually increasing trend of expression. Collectively, compared with healthy dental pulp, the expression of Cx43 in infected dental pulp was significantly upregulated (Figure 1G), suggesting that Cx43 plays a key role in the infection and inflammatory response in human dental pulp tissues.

### LPS upregulated Cx43 expression and activated Cx43-containing hemichannel activity in hDPCs

**Figure 2:**
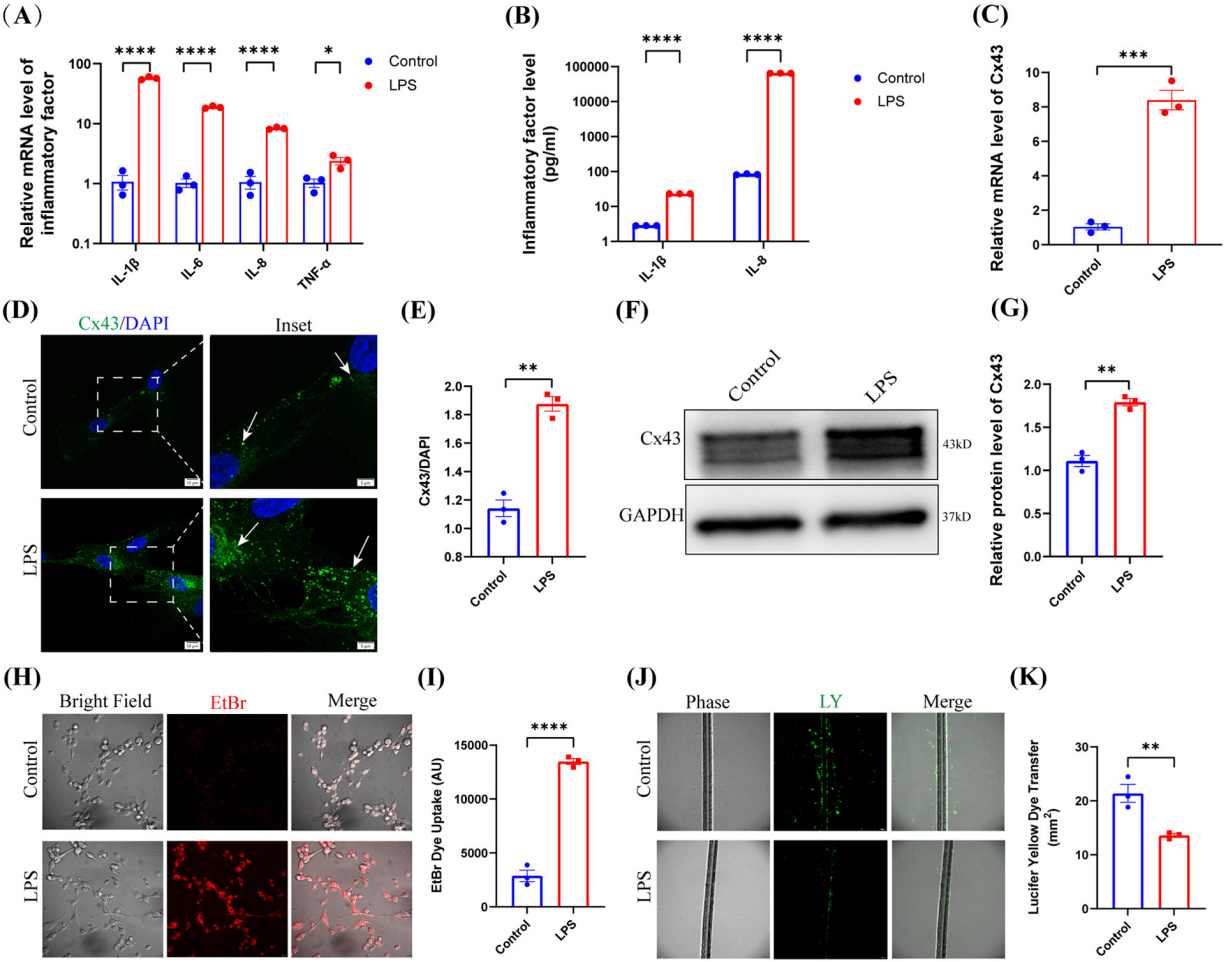
LPS upregulated Cx43 expression and activated Cx43-containing hemichannel activity. **(A)** qRT-PCR analysis of the mRNA levels of IL-1β, IL-6, IL-8, and TNF-α in hDPCs under LPS stimulation. **(B)** The protein levels of IL-1β and IL-8 were investigated by ELISA. **(C)** qRT-PCR analysis of the mRNA level of Cx43. **(D)** IF was used to investigate the fluorescence intensity and localization of Cx43 in hDPCs. **(E)** Fluorescence intensity was quantified by ImageJ software. **(F)** Equal levels of cell lysates were subjected to WB analysis to determine the protein levels of Cx43. **(G)** Protein bands were quantified by ImageJ software. **(H,I)** Analysis of the activity of Cx43 HCs by EB dye uptake and quantitative analysis. **(J,K)** Analysis of the activity of Cx43 GJs via LY scratch labeling dye tracing and quantitative analysis. All these experiments were repeated three times. *P < 0.05, **P < 0.01, ***P < 0.001, ****P < 0.0001.

Compared to the control group, LPS significantly upregulated the mRNA levels of IL-1β, IL-6, IL-8, and TNF-α within hDPCs (*P* < 0.05), as well as the protein levels of IL-1β and IL-8 (*P* < 0.0001) (Figure 2A,B). And furthermore, LPS also obviously increased the mRNA level of Cx43 in hDPCs (*P* < 0.001) (Figure 2C). We detected enhanced fluorescence intensity of Cx43 in the cytoplasm of hDPCs and on the cell membranes between adjacent cells (*P* < 0.01) (Figure 2D,E) and upregulation of the protein levels of Cx43 within hDPCs (*P* < 0.01) (Figure 2F,G).

Cx43 predominantly exerts its functionality through its constitutive channel activity. Therefore, we investigated the channel activity of Cx43 in hDPCs under LPS stimulation. Analysis demonstrated that LPS stimulated the activity of Cx43 HCs within hDPCs (Figure 2H,I), while inhibiting Cx43 GJs (Figure 2J,K), thus suggesting that Cx43 may play a role in infected hDPCs by mediating HCs but not GJs.

### Blockade of Cx43 HCs inhibited the LPS-induced TLR4-Nf-κB pathway and inflammation in hDPCs

**Figure 3:**
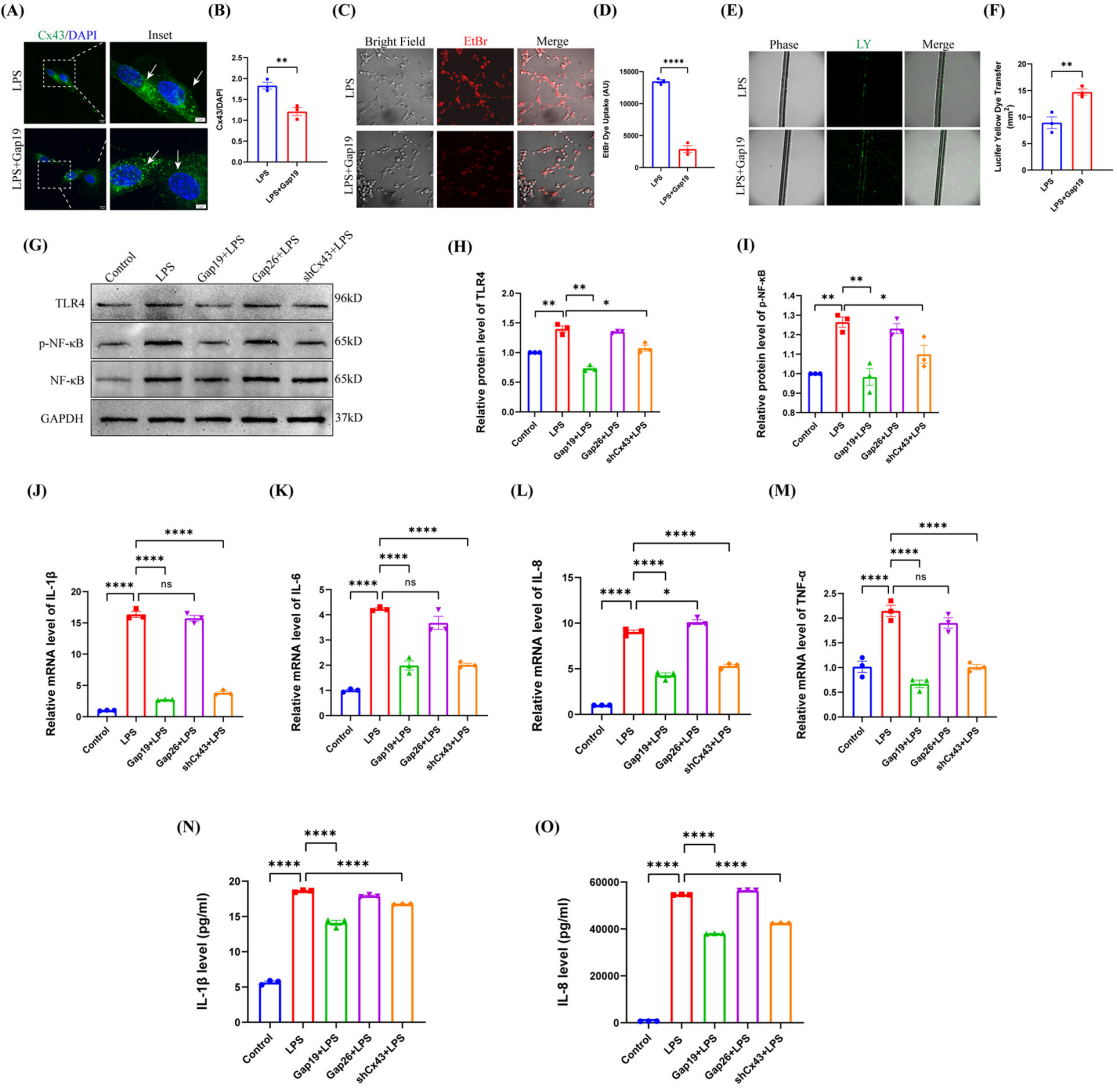
Blockade of Cx43 HCs inhibited the LPS-induced TLR4-NF-κB pathway and inflammation in hDPCs. **(A)** IF was used to investigate the fluorescence intensity of Cx43 (white arrow). **(B)** Fluorescence intensity was quantified by ImageJ software. **(C,D)** Analysis of the activity of Cx43 HCs by EB dye uptake and quantitative analysis. **(E,F)** Analysis of the activity of Cx43 GJs via LY scratch labeling dye tracing and quantitative analysis. **(G)** Equal levels of cell lysates were subjected to WB analysis to determine the protein levels of TLR4, NF-κB, and p-NF-κB. **(H,I)** Protein bands were quantified by ImageJ software. **(J–M)** qRT-PCR analysis of the mRNA levels of IL-1β, IL-6, IL-8, and TNF-α. **(N,O)** The protein levels of IL-1β and IL-8 were investigated by ELISA. All these experiments were repeated three times. shCx43: hDPCs transfected with shRNA-Cx43 lentiviral particles; ns, no statistical significance. *P < 0.05, **P < 0.01, ***P < 0.001, ****P < 0.0001.

In the physiological state, Cx43 GJs are normally open to maintain cellular homeostasis and perform critical physiological functions. Under pathological conditions, however, Cx43 GJs are often closed or downregulated; during this state, Cx43 HCs can be activated to release a class of endogenous molecules that are critical for the pathogenesis of inflammation (32). Therefore, to investigate the role of the channel activity of Cx43 in pulp inflammation, we treated hDPCs with 2 µM of Gap19, a specific peptide HC inhibitor that targets Cx43 (18, 33). Analysis showed that Gap19 significantly attenuated the fluorescence intensity of Cx43 in the cytoplasm and cell membrane of hDPCs induced by LPS (Figure 3A,B). Further analysis showed that Gap19 inhibited the activity of Cx43 HCs in hDPCs (Figure 3C,D) and promoted the activity of Cx43 GJs under LPS stimulation (Figure 3E,F), indicating that blockade of Cx43 HCs may partially restore the physiological functionality of hDPCs.

LPS stimulates TLR-4 on the cell membranes of DPCs and activates NF-κB signaling pathway, producing inflammatory cytokines such as IL-1β and IL-6 (34). To elucidate the role of Cx43 in the inflammatory response of hDPCs, we used Gap19 and Gap26 [a Cx43 GJ channel inhibitor (35)] to block the activity of Cx43 HCs and GJs in hDPCs, respectively; then, we compared these effects with Cx43 inhibition. Analysis showed that the blockade of Cx43 HCs significantly suppressed the protein level of TLR-4 and the phosphorylation level of NF-κB induced by LPS in hDPCs (*P* < 0.01), which there were no significant differences when compared to the inhibition of the expression of Cx43 (*P* > 0.05); the blockade of Cx43 GJs had no significant effect (*P* > 0.05) (Figure 3G–I). In addition, similar to Cx43 inhibition, the blockade of Cx43 HCs significantly inhibited the LPS-induced mRNA levels of IL-1β, IL-6, IL-8, and TNF-α (*P* < 0.0001) (Figure 3J–M), as well as the protein levels of IL-1β and IL-8 (*P* < 0.0001) (Figure 3N,O). Collectively, these results indicated that Cx43 regulates the LSP-induced inflammatory response in hDPCs by mediating the activity of HCs.

### The extracellular release of ATP induced by LPS stimulation exacerbated LPS-induced inflammation in hDPCs

Damage-associated molecular patterns (DAMPs) are endogenous danger molecules that are released from damaged or dying cells and activate the innate immune system by interacting with pattern recognition receptors (PRRs), thus promoting a pathological inflammatory response although they are known to contribute to host defense (36). ATP, acts as a DAMP and is released from the cytoplasm into the extracellular space to interact with specific purinergic P2 type receptors (P2XRs) to modulate inflammation (37). Furthermore, Cx HCs are known to mediate the extracellular release of ATP and act in concert with inflammatory cytokines to amplify the inflammasome pathway to perpetuate chronic inflammation (38–40). However, the role of ATP in LPS-induced inflammation in hDPCs remains unclear.

**Figure 4:**
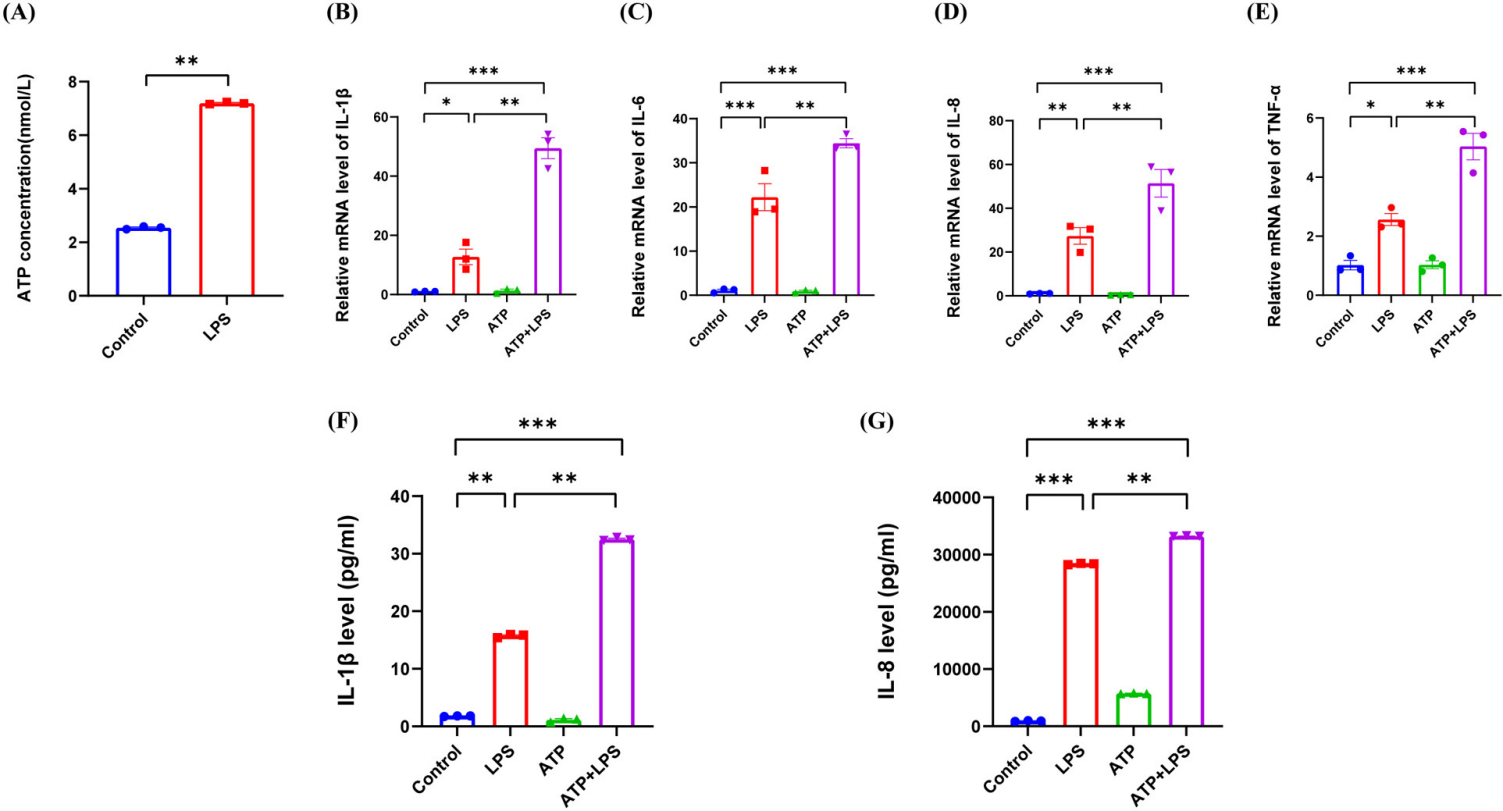
The extracellular release of ATP by LPS stimulation exacerbated the LPS-induced inflammation in hDPCs. **(A)** Analysis of the extracellular release of ATP in hDPCs with LPS stimulation, as detected by an ATP detection kit. **(B–E)** qRT-PCR analysis of the mRNA levels of IL-1β, IL-6, IL-8, and TNF-α. **(F,G)** The protein levels of IL-1β and IL-8 were investigated by ELISA. All these experiments were repeated three times. *P < 0.05, **P < 0.01, ***P < 0.001.

In the present study, we found that LPS promoted the extracellular release of ATP in hDPCs, thus resulting in a significant increase in the extracellular level of ATP (*P* < 0.01) (Figure 4A). Moreover, compared to LPS stimulation alone, additional ATP stimulation enhanced the LPS-induced mRNA levels of IL-1β, IL-6, IL-8, and TNF-α in hDPCs (*P* < 0.01) (Figure 4B–E), along with the protein levels of IL-1β and IL-8 (*P* < 0.01) (Figure 4F,G). However, ATP stimulation alone did not induce inflammation in hDPCs. Collectively, these results indicated that LPS activates the inflammatory response in hDPCs and promotes the extracellular efflux of intracellular ATP from hDPCs, while extracellular ATP further exacerbates the LPS-induced inflammatory response in hDPCs.

### The extracellular release of HMGB1 induced by LPS stimulation exacerbated LPS-induced inflammation in hDPCs

**Figure 5:**
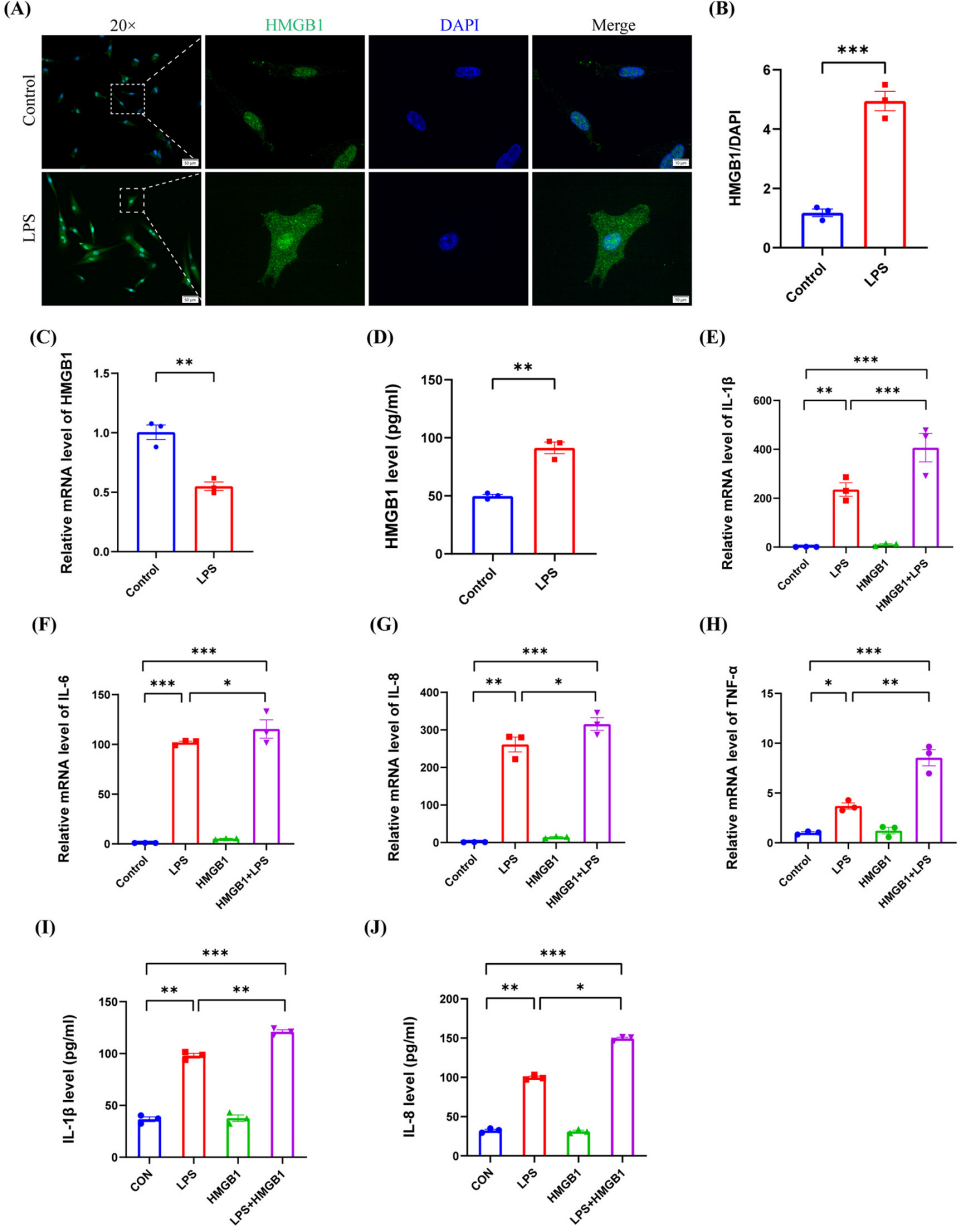
The extracellular release of HMGB1 by LPS stimulation exacerbated the LPS-induced inflammation in hDPCs. **(A)** IF was used to investigate the fluorescence intensity and localization of HMGB1 in hDPCs. **(B)** Fluorescence intensity was quantified by ImageJ software. **(C)** qRT-PCR analysis of the mRNA level of HMGB1. **(D)** The protein level of HMGB1 was investigated by ELISA. **(E–H)** qRT-PCR analysis of the mRNA levels of IL-1β, IL-6, IL-8, and TNF-α. **(I,J)** The protein levels of IL-1β and IL-8 were investigated by ELISA. All these experiments were repeated three times. *P < 0.05, **P < 0.01, ***P < 0.001.

High-mobility group box 1 (HMGB1) is another DAMP and is localized in the cell nucleus to exert critical functions in gene level. However, when released into the extracellular space, HMGB1 is known to induce inflammation by activating the NF-κB pathway by binding to TLR-2, TLR-4, and RAGE (36, 41). In this study, we found that LPS induced the expression of HMGB1 in the cytoplasm in hDPCs (*P* < 0.001) (Figure 5A,B), attenuated the mRNA transcriptional level of HMGB1 (*P* < 0.01) (Figure 5C), and upregulated the extracellular protein level of HMGB1 (*P* < 0.01) (Figure 5D), thus indicating that LPS promotes the extracellular release of HMGB1 in hDPCs. Similar to the effect of ATP, compared to LPS stimulation alone, additional HMGB1 also enhanced the LPS-induced mRNA levels of IL-1β, IL-6, IL-8, and TNF-α in hDPCs (*P* < 0.05) (Figure 5E–H), as well as the protein levels of IL-1β and 8 (*P* < 0.05) (Figure 5I,J). These data suggested that LPS not only promotes the inflammatory response of hDPCs but also leads to the extracellular release of intracellular HMGB1, while extracellular HMGB1 further exacerbates the LPS-induced inflammatory response in hDPCs.

### Cx43 HC blockade inhibited the extracellular flux of ATP and HMGB1 in hDPCs

**Figure 6:**
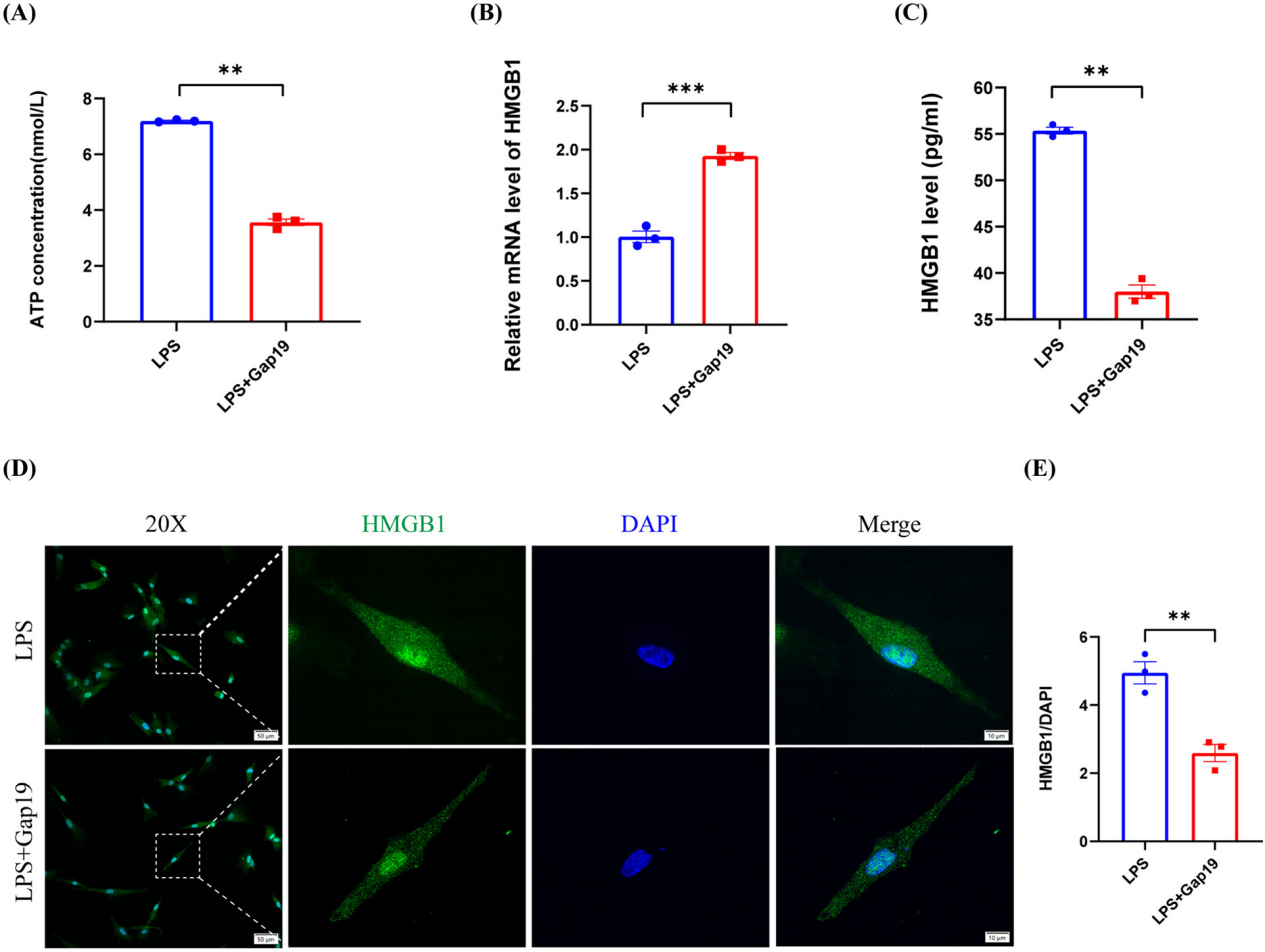
The blockade of Cx43 HCs inhibited the LPS-induced extracellular release of ATP and HMGB1 in hDPCs. **(A)** Analysis of the extracellular release of ATP, as determined by an ATP detection kit. **(B)** qRT-PCR analysis of the mRNA level of HMGB1. **(C)** The protein level of HMGB1 was investigated by ELISA. **(D)** IF was used to investigate the fluorescence intensity and localization of HMGB1 in hDPCs. **(E)** Fluorescence intensity was quantified by ImageJ software. All these experiments were repeated three times. **P < 0.01, ***P < 0.001, ****P < 0.0001.

Our previous results demonstrated that LPS induces the inflammatory response in hDPCs and simultaneously promotes the release of intracellular ATP and HMGB1 into the extracellular space. Subsequently, the extracellular ATP and HMGB1 further exacerbate the LPS-induced inflammatory response in hDPCs through autocrine or paracrine mechanisms. However, it remains unknown as to whether the extracellular efflux of ATP and HMGB1 in hDPCs is associated with Cx43 HCs. To investigate the relationship between DAMPs and Cx43 HCs in hDPCs, we blocked the activity of Cx43 HC with Gap19 to investigate the extracellular release of ATP and HMGB1. Analysis showed that the blockade of Cx43 HCs with Gap19 significantly attenuated the LPS-induced extracellular release of ATP in hDPCs (*P* < 0.01) (Figure 6A), and significantly increased the mRNA transcription level of HMGB1 in hDPCs (*P* < 0.001) (Figure 6B), whereas suppressed the extracellular protein level of HMGB1 (*P* < 0.01) (Figure 6C). Moreover, IF results showed that the blockade of Cx43 HCs inhibited the LPS-induced the expression of HMGB1 in the cytoplasm in hDPCs (Figure 6D,E). Collectively, these results demonstrated that LPS activates the inflammatory response in hDPCs, while stimulating the activity of Cx43 HCs to promote the extracellular release of ATP and HMGB1. Subsequently, the extracellular ATP and HMGB1 further exacerbate the LPS-induced inflammatory response of hDPCs. The blockade of Cx43 HCs attenuates the pro-inflammatory effects of LPS by inhibiting the extracellular efflux of ATP and HMGB1 in hDPCs.

## Discussion

In this study, we first detected the expression profile of Cx43 in normal teeth, superficial/intermediate caries, deep caries, and pulpitis, and confirmed that Cx43 plays a key role in the infection and inflammatory response of dental pulp tissues. Subsequently, we performed *in vitro* studies to demonstrate that LPS activates Cx43-containing HCs then mediate the extracellular release of ATP and HMGB1 to exacerbate the LPS-induced inflammatory response in hDPCs.

Previous research showed that Cx43 is only expressed in the odontoblast layer in healthy teeth (27). During the early stages of pulp infection, there is a transient, significant, and orderly increase in the expression levels of Cx43 at the site of infection (29). Our analysis showed that the expression levels of Cx43 were gradually upregulated as the degree of infection increased. We also found that Cx43 was expressed not only in the odontoblast layer, but also in the cell-rich zone and in the pulp proper below the infection, thus suggesting that the functionality of Cx43 is activated in deep pulpal tissues to participate in the pulpal infection and inflammatory response.

Cx43 forms HCs and can dock with HCs on adjacent cells to form GJs, thus promoting intercellular and intracellular-extracellular communication in various tissues of human body, including the heart, nervous system, and vascular system (42). Nevertheless, although Cx43 GJ and HC proteins coexist in the plasma membrane, they are not usually activated at the same time. Instead, these proteins exhibit their respective functions independently under specific conditions or states, a discovery first made by Retamal in 2007 (43). In the present study, we found that LPS upregulated the expression of Cx43 in hDPCs, with expression localized to the cell membrane, thus suggesting that the channel activity of Cx43 may be involved in the LPS-induced inflammatory response in hDPCs. Usually, Cx43 GJs are activated under physiological conditions and remain closed under pathological states; in contrast, Cx43 HCs are activated under pathological states (32). However, previous study reported that LPS can activate GJs, thus permitting the spread of intracellular infection and toxic signals to neighboring cells and the extracellular space in the CNS (44), thus suggesting that both Cx43 GJs and HCs may be involved in the pathological processes of tissues and cells. Our results showed that LPS stimulated the activity of Cx43 HCs in hDPCs and inhibited the GJs, thus indicating a potential role of Cx43 HCs in the infection and inflammation of dental pulp tissues.

In infectious diseases, Cx43 exerts functionality in different tissues and cells but does so in different ways. Previous research showed that Cx43 GJs are enhanced to activate the inflammatory response in macrophages infected by mycobacterium tuberculosis (45), while Cx43 HCs are implicated in certain diseases, including diabetic retinopathy, skin disease, kidney disease and neurological disease (39, 46–48). To further determine the channel activity of Cx43 during the inflammatory process of hDPCs, we used specific channel inhibitors to block Cx43 HCs or GJs, respectively. Our results showed that the blockade of Cx43 HCs inhibited the LPS-induced TLR4-NF-κB signaling pathway and inflammatory response in hDPCs, rather than the GJs. Collectively, these results suggested that Cx43 HCs are involved in the infection and inflammation of dental pulp tissue.

ATP is a crucial energy molecule for cellular metabolism. Under physiological conditions, the extracellular levels of ATP are low and serve as a signaling molecule for a variety of biological activities. However, during inflammation, ATP is released from the cytoplasm into the extracellular space, thus resulting in a concentration that is more than 100-fold higher than that under a normal physiological state (49). Subsequently, ATP acts on P2XRs to activate signaling pathways, such as the NF-κB pathway, thus exacerbating the inflammatory response and causing tissue damage (50–52). Cx43 HCs have also been shown to open to allow for the release of ATP, demonstrated in a range of cell types, including Cx43 transfected C6 cells and polymorphonuclear leukocytes (53–55). However, the relationship between ATP and Cx43 HCs in the LPS-induced inflammation of hDPCs has yet to be fully elucidated. In the present study, we found that LPS promoted the extracellular release of ATP in hDPCs, and that this extracellular ATP exacerbated the LPS-induced inflammatory response, thus suggesting that ATP may also promote inflammation in hDPCs via similar autocrine/paracrine pathways. The blockade of Cx43 HCs significantly reduced the LPS-induced extracellular release of ATP, thus indicating that Cx43 HCs represent a key pathway for the extracellular release of ATP in hDPCs. This finding is consistent with previous studies in diabetic retinopathy, traumatic spinal cord injury, and EAhy 926 human endothelial cells (39, 56, 57).

HMGB1 plays a role in stabilizing the structure of nucleosomes and regulating gene transcription. Under physiological conditions, HMGB1 is stably expressed primarily in the cell nucleus, as observed in the control group in Figure 5A of this study. Additionally, in certain situations, such as pathological states, HMGB1 can be released outside the nucleus and into the extracellular space to induce the inflammatory response (36, 58). Previous studies also detected the cytoplasmic expression of HMGB1 in inflamed pulp tissues (59), while the expression of HMGB1 was confined to the nuclei in healthy dental pulp (60, 61). In the present study, LPS induced the translocation of HMGB1 expression from the nucleus to the cytoplasm in hDPCs, thus promoting it release extracellularly. Similar to the effects of ATP, extracellular HMGB1 was also found to exacerbate the LPS-induced inflammatory response in hDPCs. These results indicated that infection promotes the extracellular release of HMGB1 in hDPCs, and that extracellular HMGB1 can act as a paracrine pro-inflammatory factor to exacerbate local inflammation. It is worth noting that the molecular weight of HMGB1 exceeds 1.5 kDa and that HMGB1 cannot be released into extracellular space through Cx43 HCs. However, our results showed that the blockade of Cx43 HCs inhibited the translocation of HMGB1 from the nucleus to the cytoplasm and its subsequent extracellular release in hDPCs. Similarly, previous research showed that the extracellular release of HMGB1 in macrophages and human endothelial cells induced by LPS could also be inhibited by Cx HC blockers (62). Some researchers believe that the opening of Cx43 HCs may trigger the fusion of Cx43 HCs with HMGB1-containing vesicles on the plasma membrane, thus dislodging the Cx43 HC-HMGB1 complex from the cytoskeleton and subsequently releasing HMGB1 into the extracellular space (32). Therefore, blocking the opening of Cx43 HCs by Gap19 may inhibit the fusion of Cx43 HCs with HMGB1-containing vesicles. However, further research is needed to verify this hypothesis.

**Figure 7:**
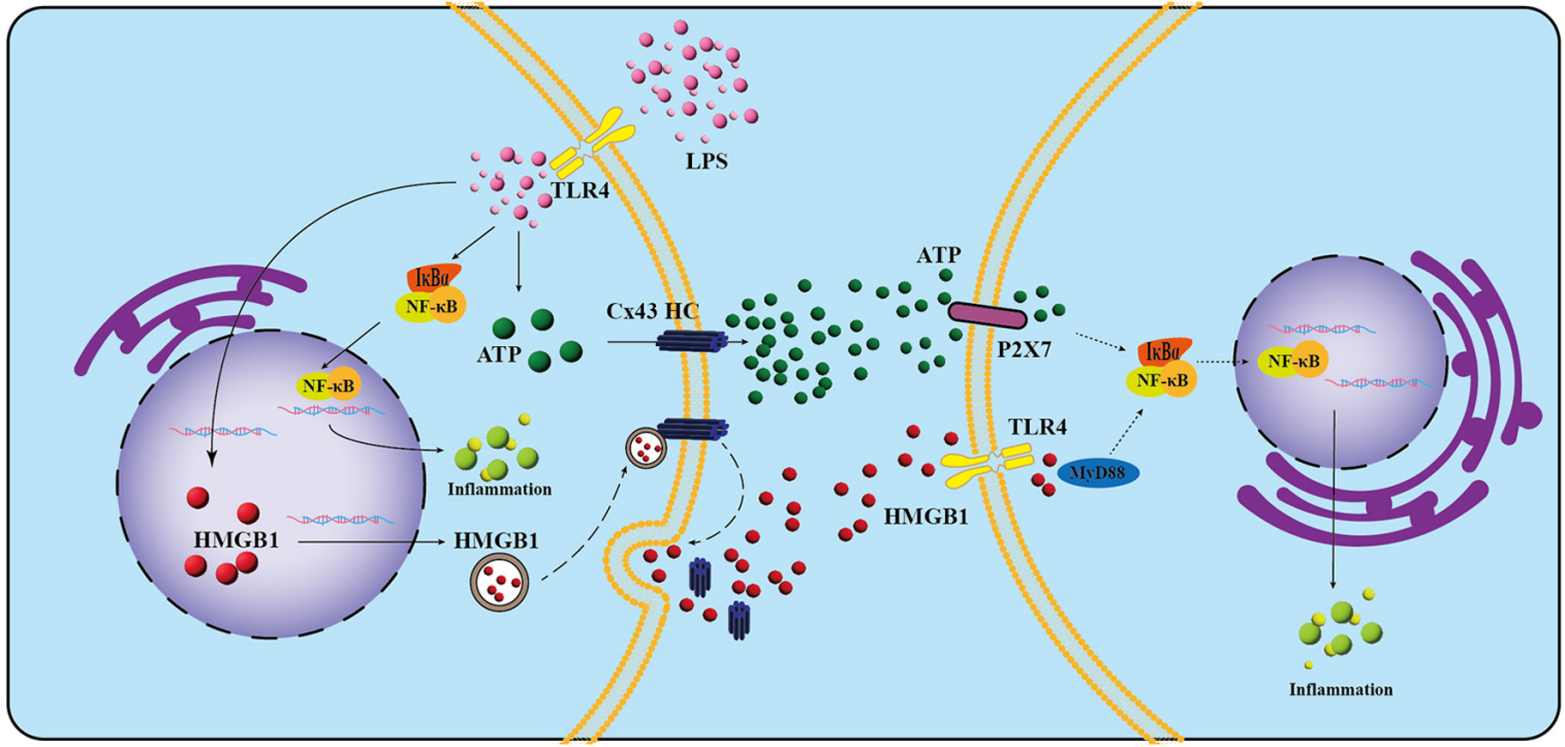
Schematic representation of the potential roles and mechanisms of Cx43 in LPS-induced inflammation in hDPCs. LPS interacts with TLR-4 on the cell membrane, leading to the phosphorylation and subsequent degradation of IκBα, which in turn facilitates the nuclear translocation of NF-κB, ultimately triggering inflammation in hDPCs. Additionally, LPS stimulates the activity of Cx43 hemichannels (HCs), promoting the extracellular release of ATP and HMGB1 through these channels. The released ATP may then bind to P2X7 receptors, further exacerbating LPS-induced inflammation via autocrine and paracrine pathways. Concurrently, the activation of Cx43 HCs by LPS may initiate their fusion with vesicles containing HMGB1 on the plasma membrane. This fusion results in the displacement of the Cx43 HC-HMGB1 complex from the cytoskeleton, allowing for the subsequent release of HMGB1 into the extracellular space. This released HMGB1 may then activate TLR-4 on neighboring cells, further promoting LPS-induced inflammation. Dashed lines in the schematic represent hypothetical inferences.

In the present study, we investigated the expression profile of Cx43 in infected dental pulp tissues and confirmed that Cx43 plays a key role in the infection and inflammation in dental pulp. When LPS promotes the inflammatory response in hDPCs, it simultaneously upregulates the expression of Cx43 and stimulates the activity of Cx43 HCs, thereby facilitating the extracellular release of ATP and HMGB1 by LPS from hDPCs. Subsequently, the extracellular ATP and HMGB1 may further exacerbate the pro-inflammatory effects of LPS via autocrine/paracrine mechanisms (Figure 7). Inhibiting the activity of Cx43 HCs attenuates the LPS-induced inflammatory response in hDPCs by reducing the extracellular release of ATP and HMGB1. Therefore, the blockade of Cx43 HC may serve as a potential strategy to attenuate the inflammatory response in infected dental pulp tissues, thereby promoting the formation of reparative dentin.

## Data Availability

The datasets presented in this study can be found in online repositories. The names of the repository/repositories and accession number(s) can be found in the article/Supplementary Material.

## References

[1] Yumoto H Hirao K Hosokawa Y Kuramoto H Takegawa D Nakanishi T The roles of odontoblasts in dental pulp innate immunity. Jpn Dent Sci Rev. (2018) 54:105–17. 10.1016/j.jdsr.2018.03.001 30128058 PMC6094490

[2] Veerayutthwilai O Byers MR Pham TT Darveau RP Dale BA. Differential regulation of immune responses by odontoblasts. Oral Microbiol Immunol. (2007) 22:5–13. 10.1111/j.1399-302X.2007.00310.x 17241164

[3] Galler KM Weber M Korkmaz Y Widbiller M Feuerer M. Inflammatory response mechanisms of the dentine-pulp complex and the periapical tissues. Int J Mol Sci. (2021) 22(3):1480. 10.3390/ijms22031480 33540711 PMC7867227

[4] Nakashima M Iohara K. Mobilized dental pulp stem cells for pulp regeneration: initiation of clinical trial. J Endod. (2014) 40:S26–S32. 10.1016/j.joen.2014.01.020 24698690

[5] Durand SH Flacher V Romeas A Carrouel F Colomb E Vincent C Lipoteichoic acid increases TLR and functional chemokine expression while reducing dentin formation in in vitro differentiated human odontoblasts. J Immunol. (2006) 176:2880–7. 10.4049/jimmunol.176.5.2880 16493045

[6] Khorasani MMY Hassanshahi G Brodzikowska A Khorramdelazad H. Role(s) of cytokines in pulpitis: latest evidence and therapeutic approaches. Cytokine. (2020) 126:154896. 10.1016/j.cyto.2019.154896 31670007

[7] Cooper PR Takahashi Y Graham LW Simon S Imazato S Smith AJ. Inflammation-regeneration interplay in the dentine-pulp complex. J Dent. (2010) 38:687–97. 10.1016/j.jdent.2010.05.016 20580768

[8] Horst OV Horst JA Samudrala R Dale BA. Caries induced cytokine network in the odontoblast layer of human teeth. BMC Immunol. (2011) 12:9. 10.1186/1471-2172-12-9 21261944 PMC3036664

[9] Hui T P A Zhao Y Wang C Gao B Zhang P EZH2, a potential regulator of dental pulp inflammation and regeneration. J Endod. (2014) 40:1132–8. 10.1016/j.joen.2014.01.031 25069920

[10] Sarfi S Azaryan E Naseri M. Immune system of dental pulp in inflamed and normal tissue. DNA Cell Biol. (2024) 43:369–86. 10.1089/dna.2024.0044 38959180

[11] Yamada M Fujino N Ichinose M. Inflammatory responses in the initiation of lung repair and regeneration: their role in stimulating lung resident stem cells. Inflamm Regen. (2016) 36:15. 10.1186/s41232-016-0020-7 29259688 PMC5725654

[12] Liu J Du J Chen X Yang L Zhao W Song M The effects of mitogen-activated protein kinase signaling pathways on lipopolysaccharide-mediated osteo/odontogenic differentiation of stem cells from the apical papilla. J Endod. (2019) 45:161–7. 10.1016/j.joen.2018.10.009 30711172

[13] He W Wang Z Luo Z Yu Q Jiang Y Zhang Y LPS promote the odontoblastic differentiation of human dental pulp stem cells via MAPK signaling pathway. J Cell Physiol. (2015) 230:554–61. 10.1002/jcp.24732 25104580

[14] Solan JL Lampe PD. Connexin43 phosphorylation: structural changes and biological effects. Biochem J. (2009) 419:261–72. 10.1042/BJ20082319 19309313 PMC2669545

[15] Noorman M van der Heyden MA van Veen TA Cox MG Hauer RN de Bakker JM Cardiac cell-cell junctions in health and disease: electrical versus mechanical coupling. J Mol Cell Cardiol. (2009) 47:23–31. 10.1016/j.yjmcc.2009.03.016 19344726

[16] Ribeiro-Rodrigues TM Martins-Marques T Morel S Kwak BR Girao H. Role of connexin 43 in different forms of intercellular communication - gap junctions, extracellular vesicles and tunnelling nanotubes. J Cell Sci. (2017) 130:3619–30. 10.1242/jcs.200667 29025971

[17] Stains JP Civitelli R. Connexins in the skeleton. Semin Cell Dev Biol. (2016) 50:31–9. 10.1016/j.semcdb.2015.12.017 26740471 PMC4779380

[18] Delvaeye T Vandenabeele P Bultynck G Leybaert L Krysko DV. Therapeutic targeting of connexin channels: new views and challenges. Trends Mol Med. (2018) 24:1036–53. 10.1016/j.molmed.2018.10.005 30424929

[19] Basheer WA Xiao S Epifantseva I Fu Y Kleber AG Hong T GJA1-20k arranges actin to guide Cx43 delivery to cardiac intercalated discs. Circ Res. (2017) 121:1069–80. 10.1161/CIRCRESAHA.117.311955 28923791 PMC5790189

[20] Laird DW Lampe PD. Therapeutic strategies targeting connexins. Nat Rev Drug Discov. (2018) 17:905–21. 10.1038/nrd.2018.138 30310236 PMC6461534

[21] Willebrords J Crespo Yanguas S Maes M Decrock E Wang N Leybaert L Connexins and their channels in inflammation. Crit Rev Biochem Mol Biol. (2016) 51:413–39. 10.1080/10409238.2016.1204980 27387655 PMC5584657

[22] Morales-Soto W Gonzales J Jackson WF Gulbransen BD. Enteric glia promote visceral hypersensitivity during inflammation through intercellular signaling with gut nociceptors. Sci Signal. (2023) 16:eadg1668. 10.1126/scisignal.adg1668 37988454 PMC10733972

[23] Yu H Cao X Li W Liu P Zhao Y Song L Targeting connexin 43 provides anti-inflammatory effects after intracerebral hemorrhage injury by regulating YAP signaling. J Neuroinflammation. (2020) 17:322. 10.1186/s12974-020-01978-z 33115476 PMC7594305

[24] Murakami S Muramatsu T Shimono M. Expression and localization of connexin 43 in rat incisor odontoblasts. Anat Embryol. (2001) 203:367–74. 10.1007/s004290100166 11411311

[25] About I Proust JP Raffo S Mitsiadis TA Franquin JC. In vivo and in vitro expression of connexin 43 in human teeth. Connect Tissue Res. (2002) 43:232–7. 10.1080/03008200290000952 12489165

[26] Ikeda H Suda H. Odontoblastic syncytium through electrical coupling in the human dental pulp. J Dent Res. (2013) 92:371–5. 10.1177/0022034513478430 23403626

[27] Yin J Xu J Cheng R Shao M Qin Y Yang H Role of connexin 43 in odontoblastic differentiation and structural maintenance in pulp damage repair. Int J Oral Sci. (2021) 13:1. 10.1038/s41368-020-00105-1 33414369 PMC7791050

[28] Li S He H Zhang G Wang F Zhang P Tan Y. Connexin43-containing gap junctions potentiate extracellular Ca(2)(+)-induced odontoblastic differentiation of human dental pulp stem cells via Erk1/2. Exp Cell Res. (2015) 338:1–9. 10.1016/j.yexcr.2015.09.008 26376117

[29] Lim WY Madden LE Becker DL. Pulpal upregulation of connexin 43 during pulpitis. Clin Oral Investig. (2021) 25:1327–35. 10.1007/s00784-020-03439-6 32623525

[30] Couve E Osorio R Schmachtenberg O. Reactionary dentinogenesis and neuroimmune response in dental caries. J Dent Res. (2014) 93:788–93. 10.1177/0022034514539507 24928097 PMC4293760

[31] Huang GT Sonoyama W Chen J Park SH. In vitro characterization of human dental pulp cells: various isolation methods and culturing environments. Cell Tissue Res. (2006) 324:225–36. 10.1007/s00441-005-0117-9 16440193

[32] Peng B Xu C Wang S Zhang Y Li W. The role of connexin hemichannels in inflammatory diseases. Biology. (2022) 11(2):237. 10.3390/biology11020237 35205103 PMC8869213

[33] Lissoni A Wang N Nezlobinskii T de Smet M Panfilov AV Vandersickel N Gap19, a Cx43 hemichannel inhibitor, acts as a gating modifier that decreases main state opening while increasing substate gating. Int J Mol Sci. (2020) 21(19):7340. 10.3390/ijms21197340 33027889 PMC7583728

[34] Sugiuchi A Sano Y Furusawa M Abe S Muramatsu T. Human dental pulp cells express cellular markers for inflammation and hard tissue formation in response to bacterial information. J Endod. (2018) 44:992–6. 10.1016/j.joen.2018.02.022 29680724

[35] Yeager M Harris AL. Gap junction channel structure in the early 21st century: facts and fantasies. Curr Opin Cell Biol. (2007) 19:521–8. 10.1016/j.ceb.2007.09.001 17945477 PMC2819411

[36] Roh JS Sohn DH. Damage-associated molecular patterns in inflammatory diseases. Immune Netw. (2018) 18(4):e27. 10.4110/in.2018.18.e27 30181915 PMC6117512

[37] Dosch M Gerber J Jebbawi F Beldi G. Mechanisms of ATP release by inflammatory cells. Int J Mol Sci. (2018) 19(4):1222. 10.3390/ijms19041222 29669994 PMC5979498

[38] Mugisho OO Rupenthal ID Paquet-Durand F Acosta ML Green CR. Targeting connexin hemichannels to control the inflammasome: the correlation between connexin43 and NLRP3 expression in chronic eye disease. Expert Opin Ther Targets. (2019) 23:855–63. 10.1080/14728222.2019.1673368 31554417

[39] Mugisho OO Green CR Kho DT Zhang J Graham ES Acosta ML The inflammasome pathway is amplified and perpetuated in an autocrine manner through connexin43 hemichannel mediated ATP release. Biochim Biophys Acta Gen Subj. (2018) 1862:385–93. 10.1016/j.bbagen.2017.11.015 29158134

[40] Roger E Chadjichristos CE Kavvadas P Price GW Cliff CL Hadjadj S Connexin-43 hemichannels orchestrate NOD-like receptor protein-3 (NLRP3) inflammasome activation and sterile inflammation in tubular injury. Cell Commun Signal. (2023) 21:263. 10.1186/s12964-023-01245-7 37770948 PMC10536814

[41] Klune JR Dhupar R Cardinal J Billiar TR Tsung A. HMGB1: endogenous danger signaling. Mol Med. (2008) 14:476–84. 10.2119/2008-00034.Klune 18431461 PMC2323334

[42] Schulz R Gorge PM Gorbe A Ferdinandy P Lampe PD Leybaert L. Connexin 43 is an emerging therapeutic target in ischemia/reperfusion injury, cardioprotection and neuroprotection. Pharmacol Ther. (2015) 153:90–106. 10.1016/j.pharmthera.2015.06.005 26073311 PMC4599355

[43] Retamal MA Froger N Palacios-Prado N Ezan P Saez PJ Saez JC Cx43 hemichannels and gap junction channels in astrocytes are regulated oppositely by proinflammatory cytokines released from activated microglia. J Neurosci. (2007) 27:13781–92. 10.1523/JNEUROSCI.2042-07.2007 18077690 PMC6673621

[44] Castellano P Eugenin EA. Regulation of gap junction channels by infectious agents and inflammation in the CNS. Front Cell Neurosci. (2014) 8:122. 10.3389/fncel.2014.00122 24847208 PMC4023065

[45] Lu Y Wang XM Yang P Han L Wang YZ Zheng ZH Effect of gap junctions on RAW264.7 macrophages infected with H37Rv. Medicine. (2018) 97:e12125. 10.1097/MD.0000000000012125 30170447 PMC6392813

[46] Cocozzelli AG White TW. Connexin 43 mutations lead to increased hemichannel functionality in skin disease. Int J Mol Sci. (2019) 20(24):6186. 10.3390/ijms20246186 31817921 PMC6940829

[47] Chen Y Wang L Zhang L Chen B Yang L Li X Inhibition of connexin 43 hemichannels alleviates cerebral ischemia/reperfusion injury via the TLR4 signaling pathway. Front Cell Neurosci. (2018) 12:372. 10.3389/fncel.2018.00372 30386214 PMC6199357

[48] Price GW Chadjichristos CE Kavvadas P Tang SCW Yiu WH Green CR Blocking connexin-43 mediated hemichannel activity protects against early tubular injury in experimental chronic kidney disease. Cell Commun Signal. (2020) 18:79. 10.1186/s12964-020-00558-1 32450899 PMC7249671

[49] Fodor P White B Khan R. Inflammation-the role of ATP in pre-eclampsia. Microcirculation. (2020) 27:e12585. 10.1111/micc.12585 31424615

[50] Diezmos EF Bertrand PP Liu L. Purinergic signaling in gut inflammation: the role of connexins and pannexins. Front Neurosci. (2016) 10:311. 10.3389/fnins.2016.00311 27445679 PMC4925662

[51] Adinolfi E Giuliani AL de Marchi E Pegoraro A Orioli E Di Virgilio F. The P2X7 receptor: a main player in inflammation. Biochem Pharmacol. (2018) 151:234–44. 10.1016/j.bcp.2017.12.021 29288626

[52] Liu JP Liu SC Hu SQ Lu JF Wu CL Hu DX ATP ion channel P2X purinergic receptors in inflammation response. Biomed Pharmacother. (2023) 158:114205. 10.1016/j.biopha.2022.114205 36916431

[53] Eltzschig HK Eckle T Mager A Kuper N Karcher C Weissmuller T ATP release from activated neutrophils occurs via connexin 43 and modulates adenosine-dependent endothelial cell function. Circ Res. (2006) 99:1100–8. 10.1161/01.RES.0000250174.31269.70 17038639

[54] Kang J Kang N Lovatt D Torres A Zhao Z Lin J Connexin 43 hemichannels are permeable to ATP. J Neurosci. (2008) 28:4702–11. 10.1523/JNEUROSCI.5048-07.2008 18448647 PMC3638995

[55] Prieto-Villalobos J Lucero CM Rovegno M Gomez GI Retamal MA Orellana JA. SARS-CoV-2 spike protein S1 activates Cx43 hemichannels and disturbs intracellular Ca(2+) dynamics. Biol Res. (2023) 56:56. 10.1186/s40659-023-00468-9 37876016 PMC10598968

[56] Saez JC Contreras-Duarte S Gomez GI Labra VC Santibanez CA Gajardo-Gomez R Connexin 43 hemichannel activity promoted by pro-inflammatory cytokines and high glucose alters endothelial cell function. Front Immunol. (2018) 9:1899. 10.3389/fimmu.2018.01899 30158937 PMC6104120

[57] Huang C Han X Li X Lam E Peng W Lou N Critical role of connexin 43 in secondary expansion of traumatic spinal cord injury. J Neurosci. (2012) 32:3333–8. 10.1523/JNEUROSCI.1216-11.2012 22399755 PMC3569730

[58] Yang H Wang H Andersson U. Targeting inflammation driven by HMGB1. Front Immunol. (2020) 11:484. 10.3389/fimmu.2020.00484 32265930 PMC7099994

[59] Wang X Sun H Hu Z Mei P Wu Y Zhu M. NUTM2A-AS1 silencing alleviates LPS-induced apoptosis and inflammation in dental pulp cells through targeting let-7c-5p/HMGB1 axis. Int Immunopharmacol. (2021) 96:107497. 10.1016/j.intimp.2021.107497 33831808

[60] Zhang X Jiang H Gong Q Fan C Huang Y Ling J. Expression of high mobility group box 1 in inflamed dental pulp and its chemotactic effect on dental pulp cells. Biochem Biophys Res Commun. (2014) 450:1547–52. 10.1016/j.bbrc.2014.07.027 25019990

[61] Tancharoen S Tengrungsun T Suddhasthira T Kikuchi K Vechvongvan N Tokuda M Overexpression of receptor for advanced glycation end products and high-mobility group box 1 in human dental pulp inflammation. Mediators Inflamm. (2014) 2014:754069. 10.1155/2014/754069 25114379 PMC4121219

[62] Li W Li J Sama AE Wang H. Carbenoxolone blocks endotoxin-induced protein kinase R (PKR) activation and high mobility group box 1 (HMGB1) release. Mol Med. (2013) 19:203–11. 10.2119/molmed.2013.00064 23835906 PMC3745600

