# Correction: Blockade of connexin43-containing hemichannel attenuates the LPS-induced inflammatory response in human dental pulp cells by inhibiting the extracellular flux of ATP and HMGB1

**DOI:** 10.3389/froh.2025.1687229

**Published:** 2025-10-08

**Authors:** Peiling Hu, Ping Long, Ruotong Li, Xiaorong Lan, Yuanpei He, Guangwen Li, Shiting Li

**Affiliations:** 1 School of Stomatology, Southwest Medical University, Lu Zhou, China; 2 Luzhou Key Laboratory of Oral & Maxillofacial Reconstruction and Regeneration, The Affiliated Stomatological Hospital, Southwest Medical University, Luzhou, China

**Keywords:** deep caries, connexin43 hemichannel, inflammatory response, dental pulp cells, DAMPs

**Error in figure/table**

Wrong content

There was a mistake in Figure 1 as published. [1. Panel 1A inadvertently displayed an incorrect tooth specimen due to a clerical error during image compilation. We have replaced it with the validated sample, consistent with original experimental records. 2. Panel 1D was misclassified as a distinct diagnostic category, duplicating the irreversible pulpitis cohort shown in Panels 1E and 1F. This panel must be deleted to resolve grouping redundancy]. The corrected Figure 1 appears below.

**Figure 1:**
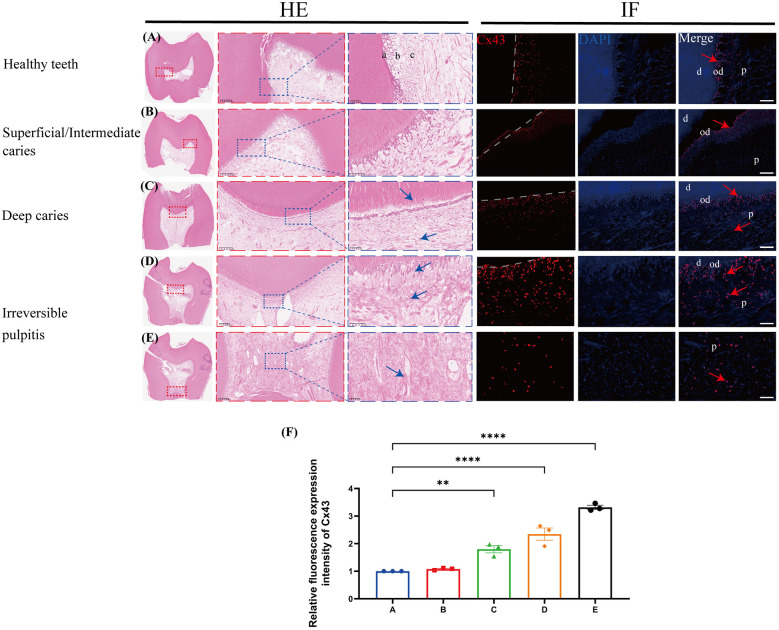
The expression profile of Cx43 in human third molars (*n* = 3/group). Cx43 (red arrow) immunofluorescence staining and quantitative analysis in infected teeth and healthy controls. Blue arrows: inflammatory cells; a/od: odontoblast layer; b: cell-rich zone; c: pulp proper; d: dentin; P: dental pulp; **(A)** Healthy teeth; **(B)** Superficial/Intermediate caries; **(C)** Deep caries; **(D,E)** Irreversible pulpitis; scale bar: 20 µm; **(F)** Quantitative fluorescence analysis of Cx43 in various groups of dental pulp. **P < 0.01, ****P < 0.0001.

Figure/table caption

There was a mistake in the caption of Figure 1 as published. [The published caption incorrectly described Panel 1A due to inadvertent specimen misidentification and erroneously included Panel 1D as a distinct diagnostic category, duplicating the irreversible pulpitis cohort. These inaccuracies necessitated panel replacement (1A), deletion (1D), re-sequencing of subsequent panels, and comprehensive legend revision]. The corrected caption of Figure 1 appears below.

“[Figure 1. The expression profile of Cx43 in human third molars (*n* = 3/group). Cx43 (red arrow) immunofluorescence staining and quantitative analysis in infected teeth and healthy controls. Blue arrows: inflammatory cells; a/od: odontoblast layer; b: cell-rich zone; c: pulp proper; d: dentin; P: dental pulp; (A) Healthy teeth; (B) Superficial/Intermediate caries; (C) Deep caries; (D,E) Irreversible pulpitis; scale bar: 20 µm; (F) Quantitative fluorescence analysis of Cx43 in various groups of dental pulp. **P < 0.01, ****P < 0.0001.]”

The original version of this article has been updated.

