# Supplementary material for: Blockade of connexin43-containing hemichannel attenuates the LPS-induced inflammatory response in human dental pulp cells by inhibiting the extracellular flux of ATP and HMGB1
Source: Front Oral Health. 2024 Dec 2;5:1496819. doi: 10.3389/froh.2024.1496819 (PMC11646852; doi:10.3389/froh.2024.1496819)
Supplement: Supplementary file 1 [file Table1.docx]

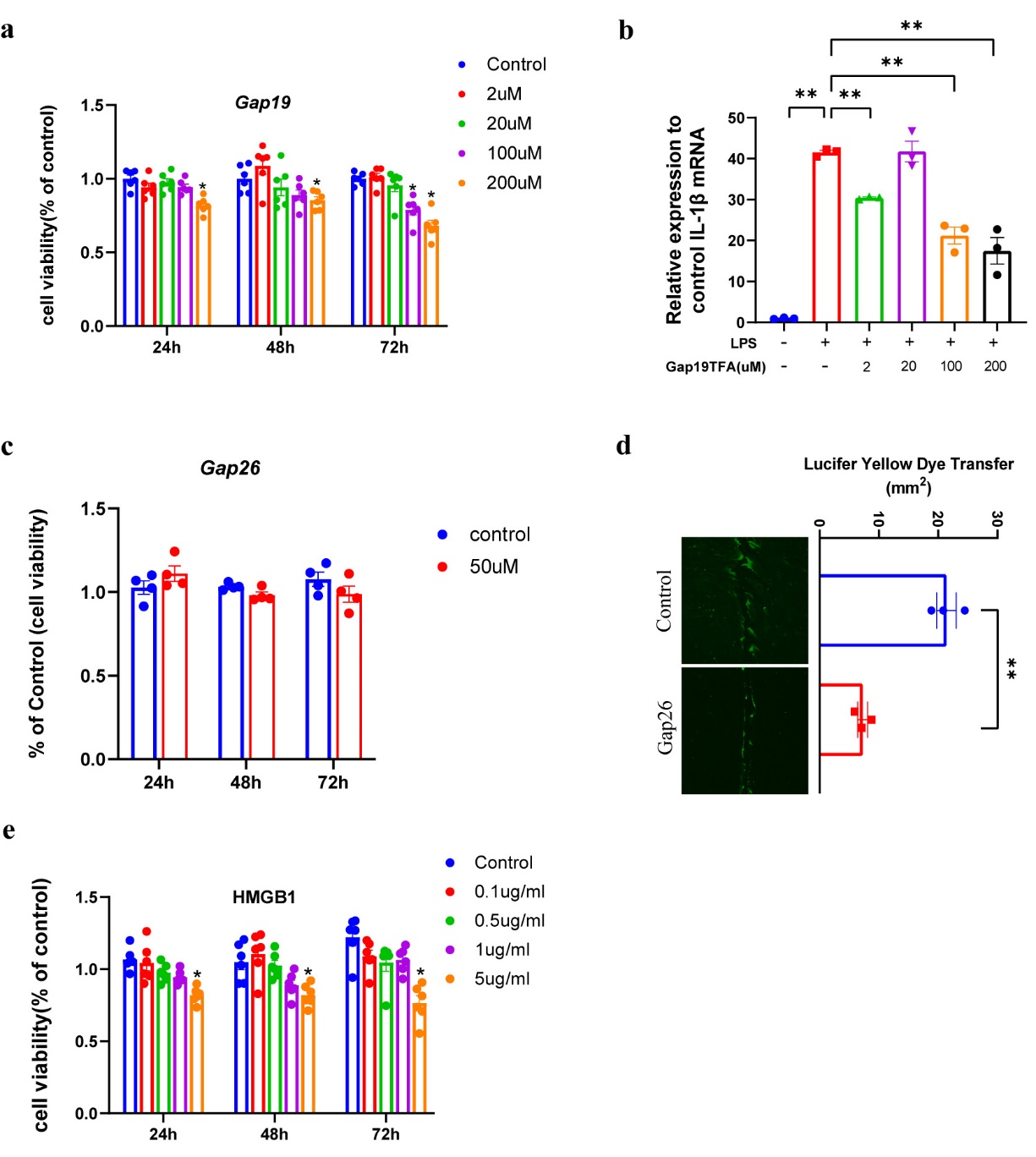


Fig. S1. Concentration screening of Gap19, Gap26, and HMGB1. **a** CCK8 assays were used to screen the effect of different concentrations of Gap19 on the viability of hDPCs. **b** qRT-PCR analysis of the mRNA expression of IL-1β in hDPCs treated with different concentrations of Gap19. Analysis led to 2 µM of Gap19 being selected for subsequent experiments. **c** Analysis of the effect of 50 µM of Gap26 on the viability of hDPCs, as determined by CCK8 assays. **d** Analysis of the effect of 50 µM of Gap26 on the channel activity of Cx43 GJs in hDPCs by LY scratch labeling dye tracing experiments. From these results, 50 µM of Gap26 was selected for subsequent experiments. **e** CCK8 assays were used to investigate the effect of different concentrations of HMGB1 on the viability of hDPCs. Analysis identified 1 µg/ml of HMGB1 as the optimum concentration for subsequent experiments. **P*＜0.05；***P* < 0.01.


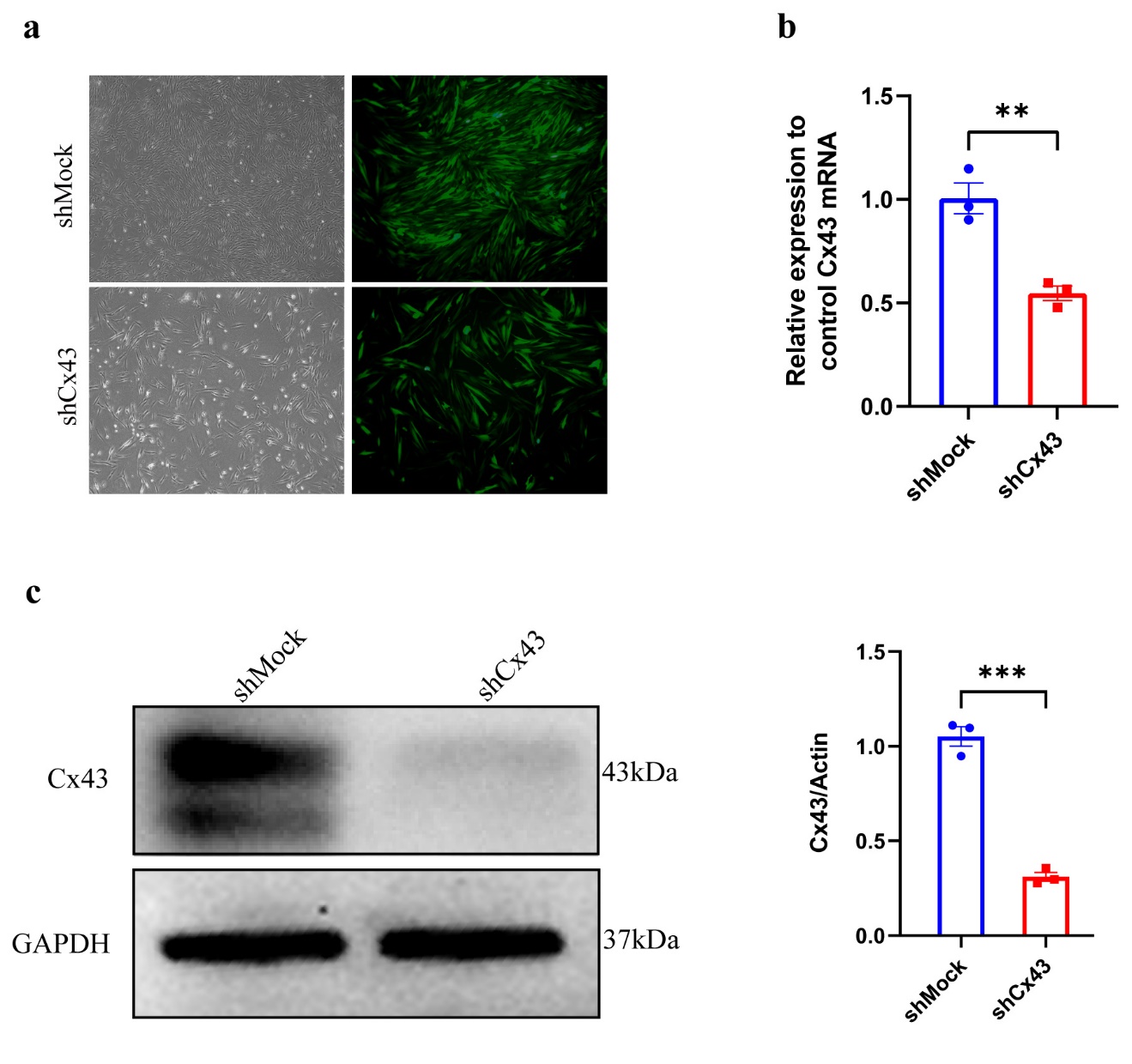


Fig. S2. The construction of lentiviral vectors to silence Cx43. **a** The fluorescence staining of Cx43 following lentiviral infection. **b** qRT-PCR analysis of the mRNA expression of Cx43. **c** Equal levels of cell lysates were subjected to WB analysis to determine the protein expression level of Cx43. Subsequently, the protein bands were quantified by ImageJ software. shCx43: Cx43 inhibition; shMock: negative control; ***P* < 0.01, ****P* < 0.001.
